# Insufficient GDF15 expression predisposes women to unexplained recurrent pregnancy loss by impairing extravillous trophoblast invasion

**DOI:** 10.1111/cpr.13514

**Published:** 2023-06-04

**Authors:** Chunzi Lyu, Tianxiang Ni, Yaqiu Guo, Tingting Zhou, Zi‐Jiang Chen, Junhao Yan, Yan Li

**Affiliations:** ^1^ Center for Reproductive Medicine Shandong University Jinan Shandong China; ^2^ Key Laboratory of Reproductive Endocrinology of Ministry of Education Shandong University Jinan Shandong China; ^3^ Department of Anesthesiology Jinan Maternal and Child Health Hospital Jinan Shandong China; ^4^ Medical Integration and Practice Center Shandong University Jinan Shandong China; ^5^ Lead Contact

## Abstract

Insufficient extravillous trophoblast (EVT) invasion during early placentation has been shown to contribute to recurrent pregnancy loss (RPL). However, the regulatory factors involved and their involvement in RPL pathogenesis remain unknown. Here, we found aberrantly decreased growth differentiation factor 15 (GDF15) levels in both first‐trimester villous and serum samples of unexplained recurrent pregnancy loss (URPL) patients as compared with normal pregnancies. Moreover, *GDF15* knockdown significantly reduced the invasiveness of both HTR‐8/SVneo cells and primary human EVT cells and suppressed the Jagged‐1 (JAG1)/NOTCH3/HES1 pathway activity, and JAG1 overexpression rescued the invasion phenotype of the *GDF15* knockdown cells. Induction of a lipopolysaccharide‐induced abortion model in mice resulted in significantly reduced GDF15 level in the placenta and serum, as well as increased rates of embryonic resorption, and these effects were reversed by administration of recombinant GDF15. Our study thus demonstrates that insufficient GDF15 level at the first‐trimester maternal–foetal interface contribute to the pathogenesis of URPL by impairing EVT invasion and suppressing JAG1/NOTCH3/HES1 pathway activity, and suggests that supplementation with GDF15 could benefit early pregnancy maintenance and reduce the risk of early pregnancy.

## Graphical abstract

By performing experiments using clinical samples from healthy pregnancies and unexplained RPL (URPL) patients, immortalized human trophoblast cells (HTR‐8/SVneo) and lipopolysaccharide (LPS)‐induced abortion mouse models, we discovered that growth differentiation factor 15 (GDF15), which is derived from human trophoblasts, was downregulated in villi and maternal sera from URPL patients. In addition, GDF15 could serve as a pro‐invasive factor in human extravillous trophoblasts and upregulate JAG1/NOTCH3/HES1 pathway activity as well as rescue the embryo absorption phenotype observed in the LPS‐induced abortion mouse model. Our findings demonstrate that insufficient GDF15 levels at the first‐trimester maternal–foetal interface contribute to the pathogenesis of URPL by impairing EVT invasion and suppressing JAG1/NOTCH3/HES1 pathway activity, therefore holding promise for diagnostic and therapeutic target applications for the clinical management of URPL.

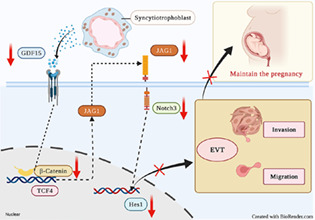

## 1 INTRODUCTION

Recurrent pregnancy loss (RPL) is defined as the failure of two or more clinically recognized pregnancies before 20 weeks of gestation.^1^, ^2^ It brings tremendous pain and suffering to prospective mothers and their families.^3^ RPL is a multifactorial disease that involves genetic, anatomical, hormonal, infectious and immune factors. Although aneuploid embryos are the most common cause of RPL,^4^ the aetiology of approximately 60% of RPL cases remains unexplained, and these cases are referred to as unexplained RPL (URPL).^2^ Research suggests that URPL is related to abnormalities at the maternal–foetal interface.^5^ However, the aetiology and underlying mechanisms of URPL remain unclear, and the clinical therapeutic regimens for URPL are limited and fraught with controversy.^6^, ^7^ Thus, a greater understanding of the aetiology and molecular mechanisms underlying UPRL is urgently needed, as it will facilitate the identification of potential molecular targets for clinical diagnosis and therapy.

The establishment and maintenance of the maternal–foetal interface during the first trimester of pregnancy is crucial for a normal pregnancy and involves complex physiological processes, including early placentation,^8^ endometrium decidualization,^9^ immune tolerance^10^ and maternal–foetal metabolic communication.^11^ During early placentation, trophoblasts differentiate into villous or extravillous trophoblasts (EVTs).^12^, ^13^ Villous trophoblasts, which include syncytiotrophoblasts (STBs) and cytotrophoblasts (CTBs), participate in nutrient exchange between the mother and foetus.^8^ EVTs that have differentiated from CTBs of the anchoring villi invade the endometrium (ingressing into the first third of the myometrium) and directly participate in remodelling of the uterine spiral artery.^13^, ^14^ Appropriate EVT invasion and migration are essential for the normal establishment of the maternal–foetal interface.^15^ It has been reported that immune responses,^16^ endoplasmic reticulum stress^17^ and oxidative stress^18^ are involved in regulating EVT invasion and migration. The identification of regulatory molecules that affect human EVT invasion during the first trimester of pregnancy would deepen our understanding of early human placentation and may facilitate the development of preventive and/or therapeutic strategies for RPL.

Growth differentiation factor 15 (GDF15), also known as macrophage inhibitory cytokine‐1, is a member of the transforming growth factor‐β (TGF‐β) superfamily.^19^ Ligands of this family bind various TGF‐β receptors, leading to recruitment and activation of SMAD family transcription factors that regulate gene expression.^20^ GDF15 is weakly expressed in most tissues but is highly expressed in the placenta during normal pregnancy.^21^ It has been linked to multiple pathological and physiological processes, including inflammation, energy metabolism, pregnancy, tumorigenesis and obesity.^19^, ^22^, ^23^, ^24^, ^25^ Several studies have reported that circulating GDF15 levels are dramatically elevated during human pregnancy, ostensibly owing to its high level of expression in placental trophoblasts.^26^, ^27^ In addition, low circulating GDF15 levels have been associated with pregnancy complications, including early pregnancy loss, preeclampsia and diabetes,^28^, ^29^ while aberrantly high circulating GDF15 levels have been associated with an increased risk of hyperemesis gravidarum.^30^ Although a predictive role of low circulating GDF15 levels for pregnancy loss from 6 to 13 weeks of gestation has been suggested,^31^ it is unclear whether reduced GDF15 level contribute to pregnancy loss.

Here, by studying clinical samples, in vitro cell models (HTR‐8/SVneo cell line and primary human EVTs), ex vivo first‐trimester villous explants and an in vivo abortion mouse model, we found that disruption of a GDF15–Jagged‐1 (JAG1) axis increased the risk of URPL by impairing EVT invasion and migration. An analysis of clinical samples from patients with URPL and women with normal pregnancies revealed decreased GDF15 protein levels in the first‐trimester serum and villi, but not the decidua, in patients with URPL. Investigations using HTR‐8/SVneo cells and primary human EVTs demonstrated that *GDF15* knockdown had no effect on cell proliferation or apoptosis but had a deleterious effect on EVT invasion by inhibiting the JAG1/NOTCH3/HES1 signalling pathway. In addition, in vivo, recombinant GDF15 partially rescued embryonic resorption in a mouse model of lipopolysaccharide (LPS)‐induced abortion. Collectively, these results demonstrate that insufficient GDF15 expression contributes to URPL pathogenesis by impairing EVT invasion, specifically by reducing JAG1/NOTCH3/HES1 signalling activity. Thus, GDF15 can be understood as a candidate diagnostic and therapeutic target for URPL clinical management.

## 2 METHODS

### 2.1 Human samples

First‐trimester human villous and decidual samples were collected from 15 normal pregnant women (controls) who underwent clinical self‐elected termination for non‐medical reasons and 15 URPL patients who underwent artificial abortion surgery for cessation of embryonic development. Patients were excluded if there was any known cause of abortion, including chromosomal abnormalities, genital malformations, diseases associated with impaired endometrial receptivity, maternal endocrine abnormalities or autoimmune disease. Tissues were immediately washed gently in cold phosphate‐buffered saline (PBS) and rapidly frozen in liquid nitrogen. Samples were stored at −80°C. Written consent was obtained from all patients included in the study.

### 2.2 Cell line culture

HTR‐8/SVneo cells were cultured in Dulbecco's modified Eagle medium (DMEM; Gibco, Foster City, CA, USA) supplemented with 10% heat‐inactivated foetal bovine serum (FBS; Becton Dickinson, Bedford, MA, USA) and 1% penicillin/streptomycin (Invitrogen, Carlsbad, CA, USA) in a humidified incubator with 5% CO_2_. HTR‐8/SVneo cells were used from passages 3–20.

### 2.3 Villous explant and primary human EVT culture

Human villous explants and primary human EVTs were obtained from normal pregnant women (controls) with elective first‐trimester pregnancy terminations, as described previously^32^ with slight modifications. First‐trimester villous samples were transferred to cold PBS containing 1% penicillin/streptomycin, and blood clots were removed as much as possible. Villi were scraped with ophthalmic scissors and cut into 2–3‐mm sections. The tissue sections were transferred to a 15‐mL centrifuge tube and allowed to stand undisturbed for 5 min, after which the supernatant was discarded. The pellets were resuspended in 1 mL of DMEM/F12 medium (Gibco) containing 10% FBS and 1% penicillin/streptomycin. Parts of the villi explants were seeded in 24‐well culture dishes pre‐coated with Matrigel (diluted 1:1, Becton Dickinson) for explant outgrowth experiments. Placental villi that successfully anchored to the Matrigel and initiated outgrowth were used for subsequent invasion studies (referred to as 0‐h samples). The explants were incubated with 50 ng/mL rhGDF15 for 24 h, and the length of the explants' outgrowth was measured at 0 h and 24 h to evaluate EVT invasion. The explants were transfected with si‐*GDF15* and a scramble control siRNA (si‐Ctrl) for 24 h, and primary human EVTs outgrown from villous pellets were digested with pancreatic enzymes and implanted into glass slides. Then immunofluorescence (IF) analysis was used to verify the knockdown efficiency.

The remaining villi pellets were explanted in 60‐mm Petri dishes pre‐coated with Matrigel (diluted 1:3) and cultured in 2 mL of 10% complete DMEM/F12 medium for 2 days. Cell culture medium was then added to a final volume of 5 mL. Primary human EVTs outgrown from villous pellets were cultured for 5–7 days to generate a sufficient number of primary EVTs, and the medium was replenished every 3 days. Adherent primary human EVTs were passaged using 0.25% trypsin (Gibco) treatment when they reached 90% confluence. Primary EVTs from passages 2–7 were used for the following experiments. Cytokeratin‐7 and human leukocyte antigen G (HLA‐G) were used to confirm the purity of the primary EVTs.

### 2.4 Cell starvation and recombinant human GDF15 treatment

HTR‐8/SVneo cells were seeded in 6‐well plates. The experimental cells were first cultured in starvation medium (0.1% FBS culture medium) for 12 h.^27^ The medium was then replaced with starvation medium containing different concentrations of recombinant human GDF15 (rhGDF15; R&D Systems Inc., Minneapolis, MN, USA), and the cells were cultured for different lengths of time (wound healing assays: 0, 24, 48 and 72 h; CCK‐8 assays: 0, 24, 48, 72 and 96 h; EdU and TUNEL assays: 24 h).

### 2.5 *GDF15* and *JAG1* small interfering RNA (siRNA) transfection

siRNAs targeting human *GDF15*, *CTNNB1* and *JAG1* and control siRNAs were purchased from GenePharma (Shanghai, China). Lipofectamine 3000 transfection reagent (Invitrogen) was used in siRNA transfection medium (DMEM supplemented with 10% FBS) according to the manufacturer's instructions. The concentration of siRNA used for cells was optimized to 50 nM.^33^ After siRNA transfection for 6 h, the transfection medium was replaced with fresh cell culture medium without penicillin and streptomycin. Proteins and mRNA were extracted 48 h after transfection to determine the knockdown efficiencies by western blotting and quantitative reverse transcription polymerase chain reaction (PCR). In villous explants, the knockdown efficiency of the target protein was confirmed by IF analysis by staining the outgrowth EVTs surrounding villous explants using the corresponding antibody. GDF15 siRNA (5′ to 3′): GCUACAAUCCCAUGGUGCUTT; AGCACCAUGGGAUUGUAGCTT. JAG1 siRNA (5′ to 3′): GGCUUUGGCUGCAAUAAGUTT; ACUUAUUGCAGCCAAAGCCTT. CTNNB1 siRNA (5′ to 3′): GCUCAUCAUACUGGCUAGUTT; ACUAGCCAGUAUGAUGAGCTT. Negative control (5′ to 3′): UUCUCCGAACGUGUCACGUTT; ACGUGACACGUUCGGAGAATT.

### 2.6 Adenoviral infection

JAG1 overexpression was achieved using an adenovirus vector, which was synthesized and packaged by HanBio Biotechnology (Shanghai, China). At 80% confluency, HTR‐8/SVneo cells and primary human EVTs were infected with an adenovirus vector directing the expression of JAG1 or an empty vector control adenovirus at a multiplicity of infection of 100:1.

### 2.7 Haematoxylin–eosin staining

Paraffin sections of human decidua and villi tissues were dehydrated in graded ethanol. Frozen sections of mouse uterus were fixed in 4% paraformaldehyde for 10 min. Paraffin sections of human decidua and villi tissues and frozen sections of mouse uterus were stained with haematoxylin for 90 s and were washed under running water for 5 min. Subsequently, they were stained with eosin for 15 s. The sections were rinsed in 95% ethanol for 1 min, dehydrated in absolute ethanol for 2 min and cleared in 2 changes of xylene for 5 min. Images were captured under a microscope (APERIO VERSA8, Leica, German).

### 2.8 Immunohistochemistry

Human decidua and villi tissues were fixed with 4% paraformaldehyde for 12 h at room temperature. After embedding the tissues in paraffin, 4‐μm sections were prepared. Xylene and a graded series of ethanol were used to deparaffinise and rehydrate the tissue sections, respectively. The tissue sections were incubated with a rabbit monoclonal antibody against human GDF15 (1:100; ab206414, Abcam, Cambridge, UK) diluted in PBS supplemented with 1% bovine serum albumin (BSA) and 0.3% Triton X‐100 (Solarbio) at 4°C overnight, followed by incubation with biotinylated goat anti‐rabbit IgG. After washing with PBS, the sections were stained with the chromogenic reagent 3,3′‐diaminobenzidine (ZSGB‐BIO, Beijing, China) for 22–25 s, followed by staining with haematoxylin. Images were captured under a microscope (Olympus, Tokyo, Japan), and the protein signals were analysed using ImageJ software (National Institute of Health, Bethesda, MD, USA). Data were analysed using SPSS 25.0 (IBM, Armonk, NY, USA) and GraphPad Prism 9 (GraphPad, San Diego, CA, USA) software.

### 2.9 Enzyme‐linked immunosorbent assay

Blood samples were collected from normal pregnant women (*n* = 21) and patients with URPL (*n* = 21) at 6–12 weeks of gestation. Blood samples were also collected from mice in the control, LPS and LPS + recombinant mouse GDF15 (rmGDF15) groups (*n* = 10 mice/group). Serum was separated by centrifugation at 1000×*g* for 15 min. Serum GDF15 concentrations were determined using a Quantikine Human GDF15 enzyme‐linked immunosorbent assay (ELISA) Kit (R&D Systems Inc., diluted 1:10) or a Quantikine Mouse/Rat GDF15 ELISA Kit (R&D Systems Inc.) according to the manufacturer's instructions.

### 2.10 RNA sequencing analysis

Human placental tissue and GDF15‐transfected HTR‐8/SVneo cells were used in this study. HTR‐8/SVneo cells were transfected with si‐*GDF15* or si‐Ctrl for 48 h. The cells were then washed with cold PBS, and RNA was extracted using TRIzol reagent (Ambion, Austin, TX, USA). RNA samples from villi from normal pregnant women (*n* = 5) and patients with URPL (*n* = 3), decidua from normal pregnant women (*n* = 9) and patients with URPL (*n* = 7), and si‐*GDF15*‐transfected HTR‐8/SVneo cells (triplicate) were sent to Novogene (Beijing, China) for RNA sequencing (RNA‐seq) analysis. Gene Ontology terms were retrieved, and Kyoto Encyclopedia of Genes and Genomes pathway analysis was performed using the Database for Annotation, Visualization and Integrated Discovery.^34^ The expression levels of relevant differentially expressed genes were verified by quantitative polymerase chain reaction (qPCR) and western blotting.

### 2.11 Western blotting

Protein was extracted from tissues or cells by lysis in radioimmunoprecipitation assay buffer containing protease and phosphatase inhibitors (Cell Signaling Technology, Danvers, MA USA), followed by centrifugation at 13,500×*g* for 38 min at 4°C. Protein samples were separated by 8% or 10% sodium dodecyl sulphate–polyacrylamide gel electrophoresis and transferred onto 0.45‐μm polyvinylidene fluoride (PVDF) membranes (Millipore, Burlington, MA, USA). After blocking with 5% non‐fat milk or 5% BSA in Tris‐buffered saline with Tween 20 (TBST) for 1 h, the PVDF membranes were incubated with primary antibodies against α‐tubulin (1:2000, #2144, CST), GDF15 (1:1000, ab206414, Abcam), JAG1 (1:1000, ab109536, Abcam), HES1 (1:300, ab108937, Abcam), non‐phosphorylated β‐catenin (1:1000, #19807, CST), β‐catenin (1:1000, #8480, CST), NOTCH 1–3 (1:1000, #3608, #5732, #5276, CST) or transcription factor 4 (TCF4; 1:1000, #2565, CST) at 4°C overnight. All antibodies were diluted with QuickBlock™ primary antibody diluent (Beyotime Biotechnology, Shanghai, China), followed by incubation with a goat anti‐rabbit secondary antibody (1:3000) in TBST at room temperature for 1 h. Immunoblots were detected with an Enhanced Chemiluminescent HRP Substrate Kit (Millipore). The protein bands were analysed using ImageJ software. α‐Tubulin was used as the control. Goat anti‐rabbit IgG was obtained from Proteintech (Rosemont, IL, USA). Antibodies against α‐tubulin, non‐phosphorylated β‐catenin, β‐catenin, TCF4 and NOTCH1‐3 were obtained from Cell Signaling Technology. The anti‐HES1, anti‐Jagged‐1 and anti‐GDF15 antibodies were purchased from Abcam.

### 2.12 Co‐immunoprecipitation

Cells were collected and lysed for 5 min on ice using Pierce IP Lysis Buffer (Pierce, Waltham, MA, USA) supplemented with protease and phosphatase inhibitor cocktail (Cell Signaling Technology) according to the manufacturer's instructions. The lysates were then incubated with anti‐non‐phosphorylated β‐catenin or anti‐TCF4 antibodies or IgG (Millipore) at 4°C overnight, followed by incubation with Protein A/G Magnetic Beads (Millipore) at room temperature for 30 min. The resulting immunocomplexes were washed three times and boiled for 10 min. The immunoprecipitates were then subjected to immunoblotting with specific antibodies.

### 2.13 Quantitative polymerase chain reaction

Total RNA was extracted from tissue samples and primary human EVTs using TRIzol reagent and from HTR‐8/SVneo cells using an RNA‐Quick Purification Kit (YiShan Biotech, Shanghai, China). Complementary DNA was synthesized using a PrimeScript™ RT Reagent Kit (TaKaRa, Kusatsu, Japan). mRNA expression levels were quantified using TB Green™ Premix Ex Taq™ (TaKaRa). The 2^−△△CT^ method was used to calculate the relative mRNA levels after normalization to actin mRNA levels. Gene‐specific primers were synthesized by BioSune (Shanghai, China), and their sequences are listed in Table S1.

### 2.14 Cell proliferation assays

Cell Counting Kit‐8 (CCK‐8; Solarbio) and 5‐ethynyl‐2′‐deoxyuridine (EdU) assay kits (RiboBio, Guangzhou, China) were used to determine the proliferation ability of viable cells. For CCK‐8 assays, HTR‐8/SVneo cells were seeded in 96‐well dishes at 4 × 10^3^ cells per well, with each well containing 100 μL of 10% or 0.1% complete DMEM. PBS was added around the 96‐well plate to avoid culture medium evaporation. Ten microliters of the CCK‐8 solution diluted in 100 μL of DMEM containing 10% or 1% FBS was added to the cells, and the cells were cultured in the dark for 2 h. At 0, 24, 48, 72 and 96 h, the absorbance was measured at 450 nm.

Cells were seeded in a 48‐well plate and incubated with 50 μM EdU solution for 2 h at 37°C, followed by fixation with 4% paraformaldehyde for 30 min and neutralization with 2 ng/mL glycine for 5 min. Apollo buffer was added to the culture, and 1× Hoechst was used to stain the nuclei. The cell proliferation results were visualized under a microscope (Olympus) and analysed using ImageJ software.

### 2.15 Terminal deoxynucleotidyl transferase dUTP nick‐end labelling (TUNEL) assay

HTR‐8/SVneo cells treated with rhGDF15 or siRNA‐mediated *GDF15* knockdown were seeded in 48‐well plates and fixed with 4% paraformaldehyde for 30 min. After fixation, the cell membranes were permeabilized with 1% Triton X‐100 for 5 min. A One‐Step TUNEL Apoptosis Assay Kit (KeyGEN BioTECH, Jiangsu, China) was used for the TUNEL assays, in accordance with the manufacturer's recommendations. The results of triplicate experiments were visualized under a microscope.

### 2.16 Wound‐healing assay

Cells were cultured in 6‐well dishes containing 2 mL of DMEM/F12 medium (without phenol red) with 0.1% FBS (Gibco) until a confluent cell monolayer was formed. A 200‐μL pipet tip was used to make a cell‐free zone across the cell monolayer. The cells were washed gently with PBS to remove cell debris, and DMEM/F12 medium (without phenol red) with 0.1% FBS was added. Wound migration was captured with digital photographs at specific time points using an inverted microscope (Olympus), and wound areas were calculated using ImageJ software. Each assay was performed in triplicate.

### 2.17 Invasion and migration assays

Transwell cell migration and Matrigel invasion assays were performed in 24‐well plates with an 8‐μm‐pore‐size Transwell filter insert (Corning, Inc., Corning, NY, USA) pre‐coated with or without Matrigel (diluted 1:9). HTR‐8/SVneo cells (6 × 10^4^ for migration assays; 8 × 10^4^ for invasion assays) or primary human EVTs (8 × 10^4^ for migration assays; 10 × 10^4^ for invasion assays) in medium with 0.1% FBS were seeded in the upper chamber, and medium supplemented with 10% FBS was added to the bottom chamber. After the cells were incubated for different time points, those on the bottom surface of the permeable membrane were fixed with 4% paraformaldehyde for 30 min, permeabilized with methanol for 15 min and stained with haematoxylin (Maiwei Biotech, Xiamen, China) for 30 min. All assays were performed in triplicate, and images were captured under a microscope. The number of cells that invaded the Matrigel and migrated to the bottom chamber was counted in four random fields and averaged.

### 2.18 Immunofluorescence

Primary human EVTs outgrown from villous explants were cultured on 14‐mm glass slides, fixed with 4% paraformaldehyde for 30 min and blocked with blocking buffer (10% BSA supplemented with 0.3% Triton X‐100) for 1 h. Subsequently, the EVTs were incubated with primary antibodies against human GDF15 (1:100, PA547839, Invitrogen), human cytokeratin 7 (1:100, 17513‐1‐AP, Proteintech), or HLA‐G (1:100, 11‐499‐C100, EXBIO, Vestec, Czech Republic) in PBS supplemented with 1% BSA and 0.3% Triton X‐100 at 4°C overnight and then with goat anti‐mouse or goat anti‐rabbit secondary antibodies (1:1000, Invitrogen) at room temperature for 1 h. Nuclear staining was then performed with an antifade reagent containing 4′,6‐diamidino‐2‐phenylindole (Invitrogen). Images were captured under a fluorescence microscope (Olympus).

Frozen sections of mouse uterus were fixed in 4% paraformaldehyde for 10 min and blocked with blocking buffer (10% FBS supplemented with 0.1% Triton X‐100) for 1 h at room temperature. The sections were incubated with primary anti‐cytokeratin 7 antibody (1:100, ab181598, Abcam) overnight at 4°C in a humid chamber. After washing three times with washing buffer (1% FBS supplemented with 0.1% Triton X‐100), the sections were incubated with goat anti‐rabbit IgG (H + L) highly cross‐adsorbed secondary antibody (Alexa Fluor™ Plus 488; 1:1000, A32731TR, Invitrogen). After three rinses with washing buffer, sections were mounted onto coverslips using Flouroshield containing DAPI (ab104139, Abcam). Panoramic images were captured under a microscope (VS120, Olympus, Tokyo, Japan).

### 2.19 LPS‐induced abortion model

Male and female C57BL/6 mice (19–21 g, 7–8 weeks old) were obtained from Beijing Vital River Laboratory Animal Technology Co. (Beijing, China). The mice were fed standard rodent chow and housed in a favourable rodent living environment. Two female mice were mated with one male mouse, and pregnancy was preliminarily confirmed by the presence of a vaginal plug. The corresponding date was recorded as gestational day (GD) 0.5. Pregnant mice were randomized into three groups (*n* = 10 mice/group), two of which received an intraperitoneal [i.p.] injection of LPS (0.25 mg/kg; Sigma–Aldrich, St Louis, MO, USA) at GD7.5 to establish murine models of LPS‐induced abortion, as previously described.^35^ The other group received an i.p. injection of PBS at GDF7.5 as a control treatment. Pregnant mice with LPS‐induced abortion were randomly assigned to receive an i.p. injection of rmGDF15 (0.01 mg/kg; R&D Systems Inc.) at GD7.5 and GD10.5. Embryonic tissues and blood samples were collected at GD12.5 to measure GDF15 expression levels. All mice from the three groups were euthanized with isoflurane (RWD Life Science, Shenzhen, China) at GD12.5 to determine the foetal resorption rate. Blood samples were obtained from the retro‐orbital plexus, and serum was separated for GDF15 measurements. Blood and uterus samples were obtained from all three groups. The depth of CK7+ trophoblast infiltration into the uterus of pregnant mice (ratio of L1/L2) was determined by IF analysis.^36^, ^37^

### 2.20 Statistics

Data were analysed using SPSS 25.0 and GraphPad Prism 9.0 software. Data for normally distributed variables are expressed as the mean ± standard deviation (SD). Analysis of variance was used to compare multiple groups, while unpaired or paired Student's t tests were used to analyse measured data. *p* Values <0.05 were considered statistically significant.

### 2.21 Study approval

All protocols involving human subjects and animal experiments were reviewed and approved by the Ethics Committee of the Center for Reproductive Medicine of Shandong University.

## 3 RESULTS

### 3.1 First‐trimester villi and serum from URPL patients have aberrantly low GDF15 level

**FIGURE 1:**
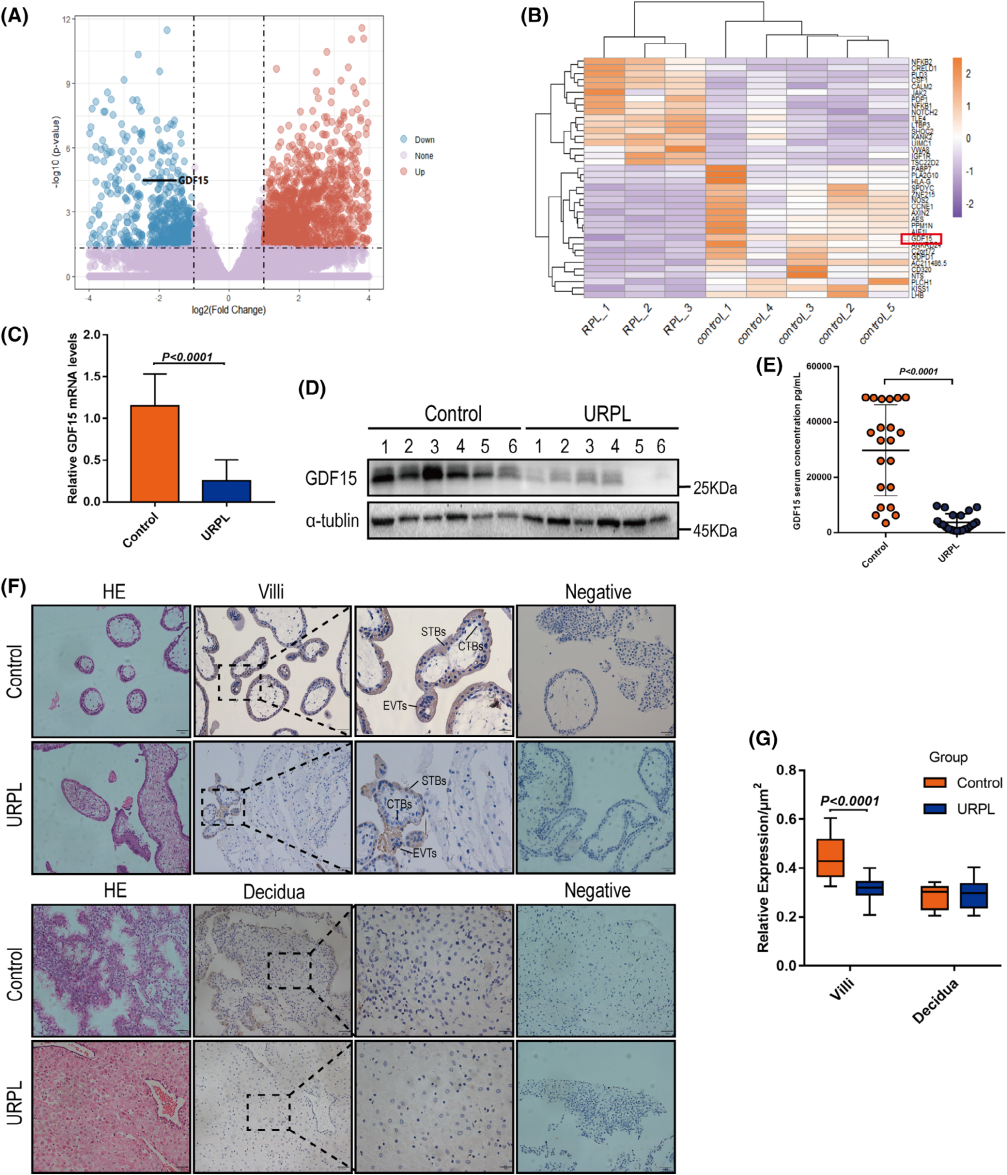
GDF15 levels in villi and serum from URPL patients. (A, B) Volcano plot of RNA sequencing data showing the most differentially expressed genes (DEGs) in URPL villi (*n* = 3) and normal pregnant (control) villi (*n* = 5). Significantly regulated genes above and below two‐fold are shown in blue and red, respectively. Red dots indicate genes with log2‐fold change >1, blue dots indicate genes with log2‐fold change <1. (C) qPCR was performed to measure the *GDF15* mRNA levels in villi from nine URPL patients and nine controls. (D) Western blotting analysis of GDF15 protein levels in villi from six URPL patients and six normal controls. (E) ELISA was performed to analyse serum GDF15 levels in patients with URPL (*n* = 21) and normal controls (*n* = 21). (F, G) Haematoxylin and eosin staining and representative immunohistochemical staining against GDF15 in villi and decidua from patients with URPL and normal controls (*n* = 15/group). EVTs, extravillous trophoblasts; CTBs, cytotrophoblasts; STBs, syncytiotrophoblasts. Data are presented as the means ± SDs. *p* < 0.01 by two‐tailed Student's *t* test.

To gain a global view of transcriptome changes in URPL villi, we carried out RNA‐seq analysis in three villi samples from URPL patients and five villi samples from women with normal pregnancies (controls; Figure 1A,B). Amongst the differentially expressed genes (*p* < 0.05), *GDF15*, *ANXA5*, *CREB5* and *TEAD4* were previously believed to be linked to pregnancy loss.^38^, ^39^, ^40^, ^41^ We found that *GDF15* was also significantly downregulated in the URPL villi (Figure 1A, B). A qPCR analysis of villi (9 URPL patients vs. 9 controls) confirmed the GDF15 expression trend detected in the RNA‐seq analysis (Figure 1C), and immunoblotting showed that GDF15 protein level was significantly decreased in URPL villi (6 URPL patients vs. 6 controls) (Figure 1D). An ELISA analysis of sera showed that URPL patients had significantly lower GDF15 level than the controls (*n* = 21/group; Figure 1E).

To identify the cell‐type‐specific expression profiles of GDF15 at the maternal‐foetal interface during the first trimester of pregnancy, we examined single‐cell transcriptome datasets (E‐MTAB‐6701 and E‐MTAB‐6678) from human placentas and decidua at 6–14 weeks of gestation and matched peripheral blood mononuclear cells.^42^ GDF15 is strongly expressed in EVT, STB and CTB cells (Figure S1). We also used immunohistochemistry to examine GDF15 levels at the human first‐trimester maternal‐foetal interface (i.e., placental villi and decidual tissues) from URPL patients and controls (*n* = 15/group). The GDF15 protein signal evident in villi (and enriched in EVTs and STBs) was significantly weaker in the URPL villi samples than in the control villi samples. There was a weak but detectable GDF15 signal in the decidua, with no obvious difference between URPL patients and controls (Figure 1F,G). These results indicate the involvement of decreased GDF15 levels in the first‐trimester villi and maternal sera in URPL pathogenesis.

### 3.2 GDF15 knockdown impairs EVT invasion and migration as examined in ex vivo and in vitro models

**FIGURE 2:**
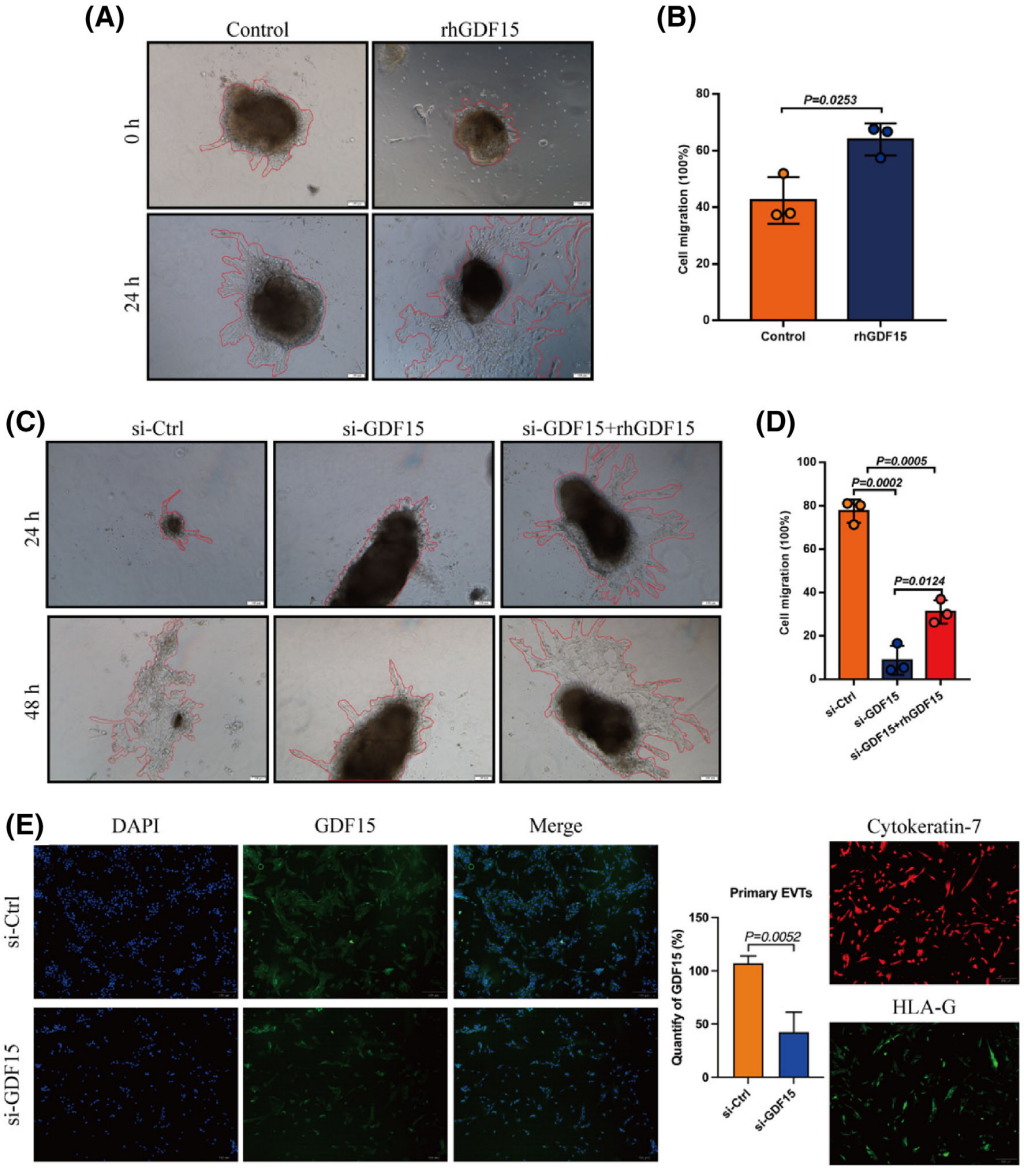
GDF15 regulates extravillous trophoblast outgrowth in villous explant cultures. (A, B) First‐trimester extravillous explants from normal pregnancies were cultured with 50 ng/mL rhGDF15 for 24 h, and images of the explants were captured under a light microscope after 0 and 24 h. Representative images are shown in the left panel, and summarized quantitative results are shown in the right panel. (C, D) Extravillous explants were transfected with non‐targeting control siRNA (siCtrl) or siRNA targeting *GDF15* (si‐GDF15) prior to treatment with or without rhGDF15 (50 ng/mL), and images of the explants were captured under a light microscope after 24 h and 48 h. The migration distance of villus tips was measured and analysed as shown in panel D. (E) Immunofluorescence staining (IF) of GDF15, cytokeratin‐7 and HLA‐G in primary EVTs outgrown from extravillous explants. The levels of GDF15 were assayed by IF (left panel). The knockdown efficiency for GDF15 protein was assessed by ImageJ software (middle panel). The purity of the primary human EVTs was examined by staining cytokeratin‐7 (red) and HLA‐G (green) (right panel). Scale bar, 100 μm. Data are presented as the means ± SD of three independent experiments. *p* < 0.01 by two‐tailed Student's *t* test.

As a secretory molecule, GDF15 has been identified as a pro‐invasive factor in multiple carcinomas.^43^, ^44^ To investigate the potential functions of GDF15 in regulating human EVT invasion ex vivo, first‐trimester placental villous explants were cultured on Matrigel‐coated dishes. Briefly, the explants incubated with recombinant human GDF15 (rhGDF15) showed significantly increased invasion ability (measured as outgrowth distances) compared to PBS‐control explants (Figure 2A,B). We also transfected explants with si‐*GDF15* or scramble control siRNA (si‐Ctrl) and performed outgrowth assays (Figure 2C,D); the knockdown efficiency of si‐GDF15 transfection in first‐trimester villous explants was assessed by immunofluorescence (Figure 2E). GDF15 knockdown resulted in significantly decreased EVT outgrowth compared with controls (*n* = 3/group). Importantly, supplementation with rhGDF15 rescued this GDF15 knockdown‐mediated defect in EVT outgrowth (Figure 2C,D). These findings support that GDF15 functions as a pro‐invasive factor in regulating human EVT invasion.

**FIGURE 3:**
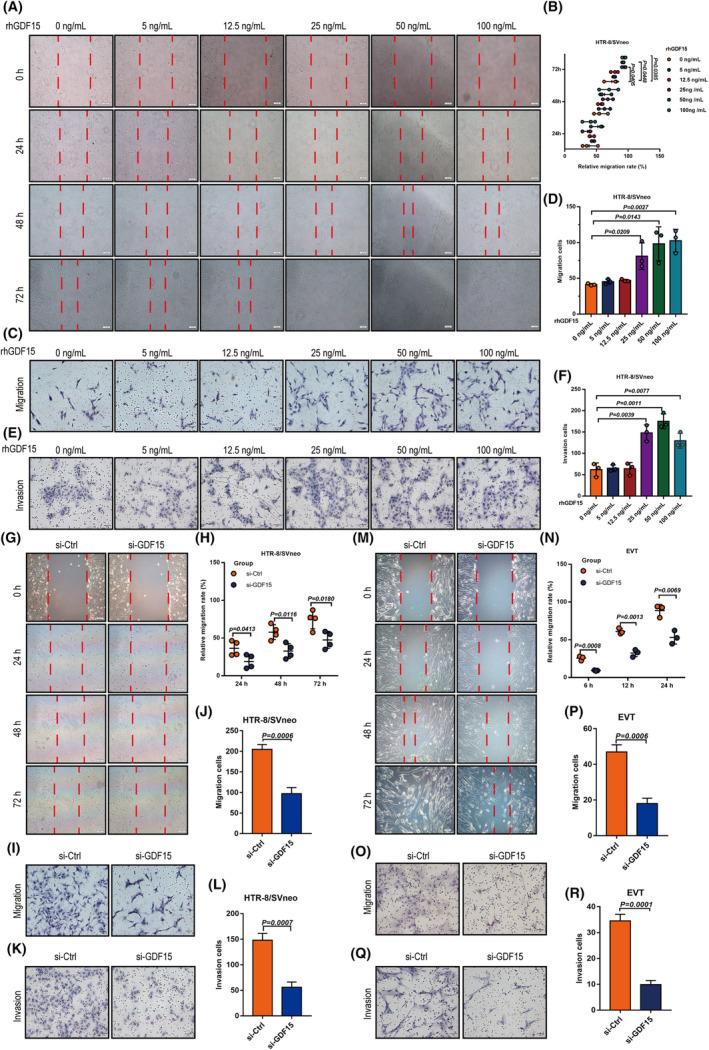
GDF15 promotes invasion and migration of extravillous trophoblasts in vitro. (A, B) HTR‐8/SVneo cells were treated with different concentrations of rhGDF15 (0–100 ng/mL) for cell migration analysis (wound‐healing assays). Representative images are presented in panel A, and the relative migration rate in each group was quantified and summarized in panel B. (C‐D) Transwell™ assays were performed to examine the migratory abilities of rhGDF15‐treated HTR‐8/SVneo cells. Representative images are presented in panel C, and the number of migrating cells in each group was quantified and summarized in panel D. (E, F) Matrigel‐coated Transwell™ assays were performed to study cell invasion in rhGDF15‐treated HTR‐8/SVneo cells. Representative images are presented in panel E, and the number of invading cells in each group was quantified and summarized in panel F. (G, H) HTR‐8/SVneo cell motility was examined with wound‐healing assays after transfection with non‐targeting control siRNA (si‐Ctrl) or siRNA targeting *GDF15* (si‐GDF15). Panel G shows representative images of the wound‐healing assay; panel H shows the summarized quantitative results of the wound‐healing assay. (I, J) HTR‐8/SVneo cell motility was examined with Transwell™ assays after transfection with si‐Ctrl or si‐GDF15. (I) The panel shows representative images of the migration assay. (J) The panel shows the summarized quantitative results of the migration assay. (K, L) HTR‐8/SVneo cell invasiveness was examined with Matrigel‐coated Transwell™ assays after transfection with or without si‐GDF15. (K) The panel shows representative images of the invasion assay. (L) The panel shows the summarized quantitative results of the invasion assay. Wound‐healing assay (M, N) and Transwell™ assay (O‐P) were transfected with si‐Ctrl or si‐GDF15. (M) The panel shows representative images of the wound‐healing assay. (N) The panel shows the summarized quantitative results of the wound‐healing assay. (O) The panel shows representative images of the migration assay. (P) The panel shows the summarized quantitative results of the migration assay. (**Q‐R**) Matrigel‐coated Transwell™ assays were performed to examine cell invasiveness after siRNA‐mediated *GDF15* knockdown in primary human EVTs. Representative images are presented in the left panel, and the number of invading cells was quantified and summarized in the right panel. Data are presented as the means ± SDs from at least three independent experiments. *p* < 0.05, and *p* < 0.01 by two‐tailed Student's *t* test.

We also examined the invasive and migratory abilities of HTR‐8/SVneo cells (Figure 3A–L) and primary human EVTs (Figure 3M–R): GDF15 increased cell invasion and migration, doing so in a dose‐dependent manner. Note that EdU, CCK‐8, and TUNEL assays showed that GDF15 did not affect cell proliferation (Figure S2A‐S2F) or apoptosis (Figure S2G‐S2J). Together, these ex vivo and in vitro findings show that GDF15 exerts pro‐migratory and pro‐invasive functions in human EVTs, suggesting that the aberrantly low GDF15 level observed at the maternal‐foetal interface could impair EVT invasion and thus contribute to URPL pathogenesis.

### 3.3 *GDF15* knockdown suppresses the JAG1/NOTCH3/HES1 signalling pathway

**FIGURE 4:**
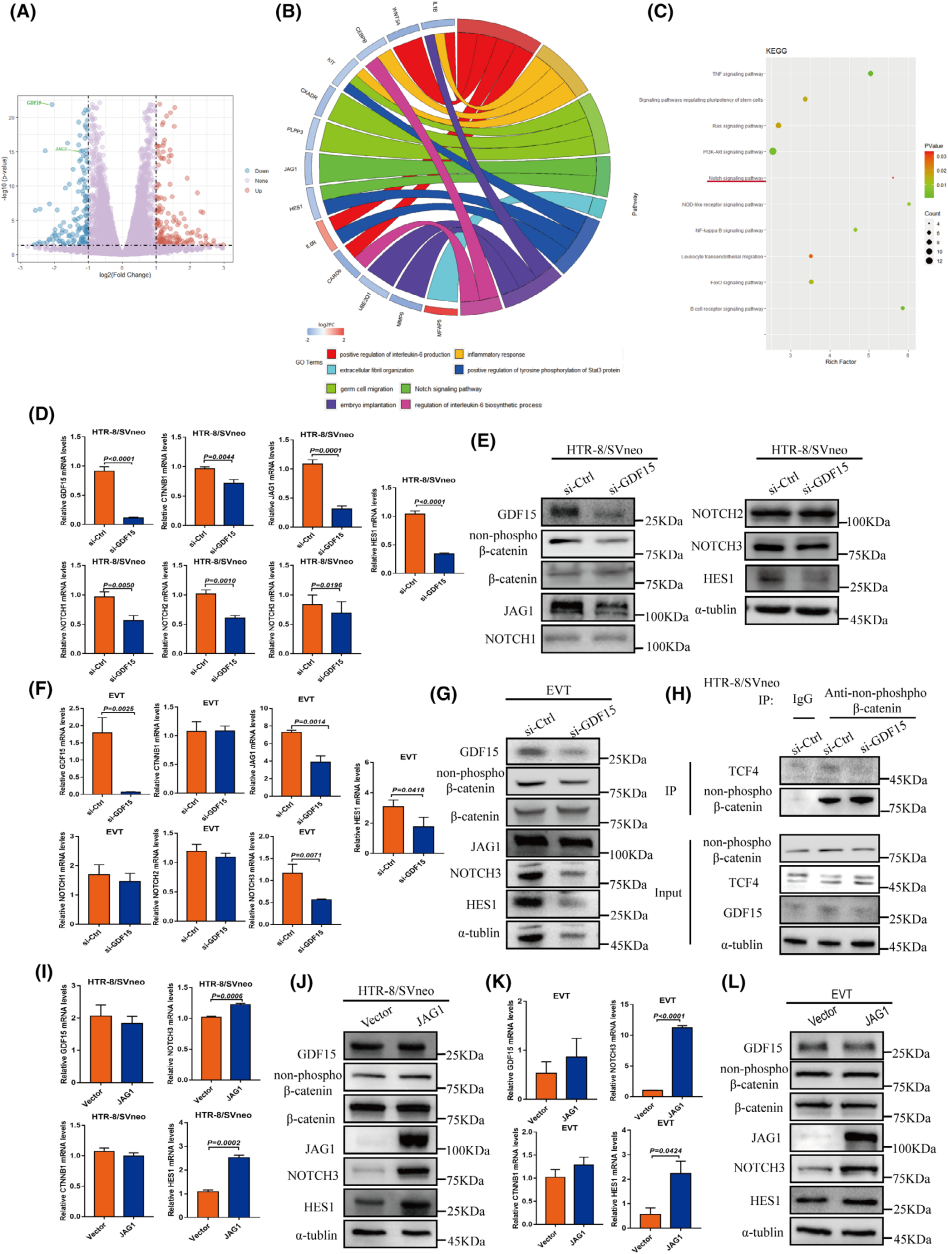
JAG1 functions downstream of GDF15 in extravillous trophoblasts. (A) Volcano map of the si‐*GDF15*‐transfected HTR‐8/SVneo cell transcriptome. *GDF15* and *JAG1* are labelled. (B) Gene Ontology (GO) term analysis. (C) Kyoto Encyclopedia of Genes and Genomes (KEGG) pathway analysis. (D) qPCR analysis of *GDF15*, *CTNNB1*, *JAG1*, *NOTCH1‐3* and *HES1* mRNA levels in HTR‐8/SVneo cells transfected with si‐*GDF15*. (E) Western blotting analysis of GDF15, β‐catenin, JAG1, NOTCH1‐3 and HES1 in HTR‐8/SVneo cells transfected with si‐*GDF15*. (F) qPCR analysis of *GDF15*, *CTNNB1*, *JAG1*, *NOTCH1‐3* and *HES1* in primary human EVTs with or without *GDF15* knockdown. (G) Western blotting analysis of GDF15, β‐catenin, JAG1, NOTCH3 and HES1 in primary human EVTs transfected with or without si‐*GDF15*. (H) The association between TCF4 and non‐phosphorylated β‐catenin was assessed by co‐IP analysis with an anti‐non‐phosphorylated β‐catenin antibody in HTR‐8/SVneo cells with or without *GDF15* knockdown. (**I**) qPCR analysis of *GDF15*, *CTNNB1*, *NOTCH3* and HES1 expression in HTR‐8/SVneo cells with or without JAG1‐expressing adenovirus infection. (J) Western blotting analysis of GDF15, β‐catenin, JAG1, NOTCH3 and HeS1 expression in HTR‐8/SVneo cells with or without JAG1‐expressing adenovirus infection. (K) qPCR analysis showed that JAG1 overexpression increased the mRNA levels of *NOTCH3* and *HES1* in primary human EVTs. (L) Western blotting analysis showed that JAG1 overexpression increased the protein levels of NOTCH3 and HES1 in primary human EVTs. Data are presented as the means ± SDs from at least three independent experiments. *p* < 0.05, and *p* < 0.01 by two‐tailed Student's *t* test.

To examine potential molecular targets of GDF15 in human trophoblasts, we used RNA‐seq to profile the transcriptomes of *GDF15* knockdown HTR‐8/SVneo cells (Figure 4A–C). The expression level of *JAG1*, which encodes the Notch ligand Jagged‐1 protein, was significantly decreased in HTR‐8/SVneo cells upon *GDF15* knockdown (Figure 4D). Notch signalling has been implicated in the promotion of trophoblast migration,^45^ and previous studies have demonstrated that disrupting JAG1 function suppresses blastocyst implantation capacity in both humans and mice.^46^

We investigated the potential impacts of GDF15 on the JAG1/NOTCH signalling pathway. Specifically, given previous reports that GDF15 impacts cell viability via β‐catenin^47^, ^48^ and considering that *JAG1* is a direct target gene of β‐catenin in colorectal cancer cells,^49^ we conducted qPCR and immunoblotting analyses of siRNA‐mediated knockdown of *GDF15* in HTR‐8/SVneo cells and found that GDF15 upregulated β‐catenin and JAG1. We performed siRNA‐mediated knockdown of *CTNNB1*, which encodes the β‐catenin protein, in HTR‐8/SVneo cells. *CTNNB1* knockdown led to decreased mRNA levels of *JAG1* and the NOTCH pathway downstream molecule *HES1* (Figure S3A). Moreover, immunoblotting showed that *GDF15* knockdown led to decreased expression levels of β‐catenin, JAG1, NOTCH3 and HES1; *GDF15* knockdown did not affect NOTCH1 or NOTCH2 levels (Figure 4E). In primary human EVTs, qPCR and immunoblotting analyses showed that *GDF15* knockdown led to significant decreases in the levels of β‐catenin, JAG1, NOTCH3 and HES1 (Figure 4F,G).

Previous studies have shown that GDF15 stimulates melanogenesis in melanocytes via β‐catenin signaling^50^ and that WNT‐β‐catenin‐T‐cell‐specific factor (TCF) signalling induces JAG1‐mediated induction of Notch signalling in gastric cancer.^51^ We conducted a co‐immunoprecipitation assay of extracts from HTR‐8/SVneo cells and found that GDF15 knockdown reduced the extent of the interaction between non‐phosphorylated β‐catenin and TCF4 (Figure 4H), suggesting that *GDF15* knockdown decreased the accumulation of β‐catenin and its recruitment to the TCF complex.

We next performed siRNA‐mediated knockdown of *JAG1* in HTR‐8/SVneo cells and observed decreased levels of NOTCH3 and HES1 but no changes in GDF15 or β‐catenin levels (Figure S3B,S3C), findings supporting that JAG1 functions downstream of GDF15. Conversely, JAG1 overexpression increased NOTCH3 and HES1 mRNA and protein levels (Figure 4I,J). The regulatory effects of JAG1 on NOTCH3 and HES1 expression were again observed in primary human EVTs transfected with si‐*JAG1* (Figure S3D,S3E) or with a vector for JAG1 overexpression (Figure 4K,L). These results suggest that *GDF15* knockdown suppresses the JAG1/NOTCH3/HES1 signalling pathway by reducing the accumulation of β‐catenin in human EVTs.

### 3.4 JAG1 mediates GDF15‐induced human EVT invasion and migration

To assess the potential functions of JAG1 as a downstream mediator of GDF15, we tested whether *JAG1* knockdown phenocopied the effects observed upon *GDF15* knockdown in HTR‐8/SVneo cells and primary human EVTs. Wound healing, migration, and invasion assays were performed in HTR‐8/SVneo cells: *JAG1* knockdown decreased HTR8/SVneo cell migration and invasion (Figure S4A‐S4C). Conversely, JAG1 overexpression increased HTR‐8/SVneo cell migration and invasion (Figure S4D‐S4F). Consistent findings were also observed for primary human EVTs, indicating that JAG1, like GDF15, functions as a pro‐invasive factor in human EVT invasion (Figure S4G‐S4I).

**FIGURE 5:**
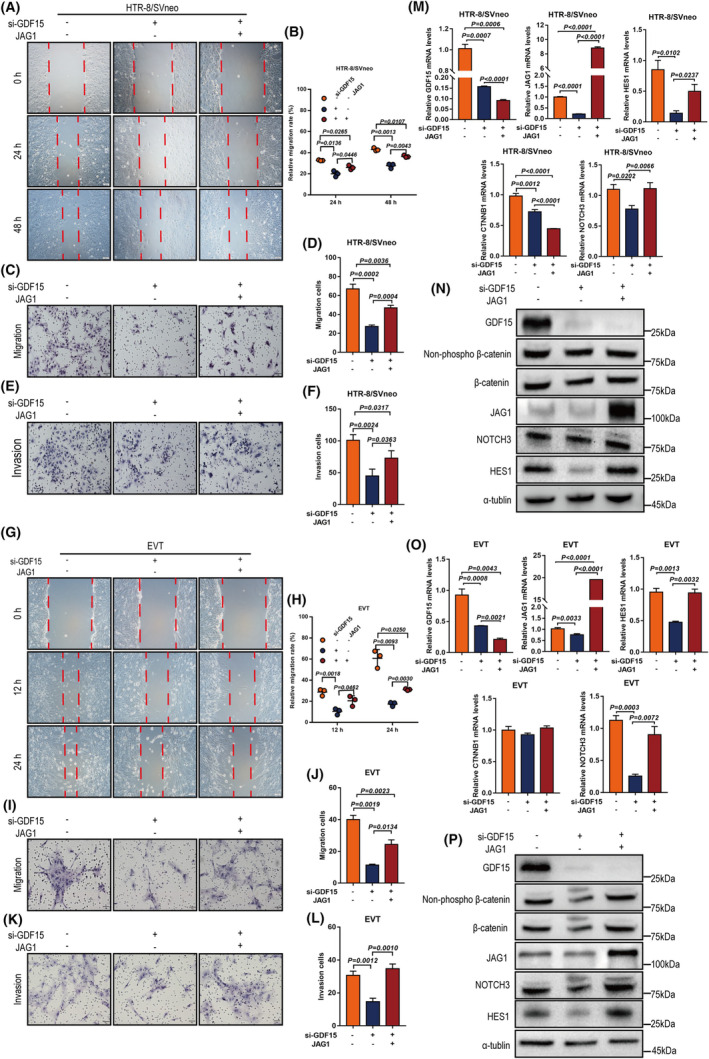
GDF15 promotes invasion and migration of extravillous trophoblasts by upregulating JAG1 expression. (A–F) HTR‐8/SVneo cells were transfected with siRNA targeting *GDF15* (si‐GDF15) prior to treatment with or without JAG1 overexpression. Cell mobility was examined with wound‐healing assays (A, B) and Transwell™ assays (C, D); cell invasiveness was examined with Matrigel‐coated Transwell™ assays (E, F). Representative images are presented in panels A, C and E, and the summarized quantitative results are presented in panels B, D and F. (G–L) Primary human EVTs were transfected with siRNA targeting *GDF15* (si‐GDF15) prior to treatment with or without JAG1 overexpression, followed by wound‐healing assays (G, H) and Transwell™ assays (I, J) of cell migratory capacity and Matrigel‐coated Transwell™ assays of cell invasiveness (K, L). Representative images are presented in panels G, I and K, and the summarized quantitative results are presented in panels H, J and L. (M) qPCR analysis of *GDF15*, *CTNNB1*, *JAG1*, *NOTCH3* and *HES1* mRNA levels in HTR‐8/SVneo cells transfected with si‐Ctrl, si‐*GDF15* or si‐*GDF15* + JAG1‐overexpression vector. (N) Western blotting analysis of GDF15, β‐catenin, JAG1, NOTCH3 and HES1 protein levels in HTR‐8/SVneo cells transfected with si‐Ctrl, si‐*GDF15* or si‐*GDF15* + JAG1‐overexpression vector. EVTs were transfected with si‐Ctrl, si‐*GDF15* or si‐GDF15 + JAG1‐overexpression vector for 48 h. (O) The mRNA levels of *GDF15*, *CTNNB1*, *JAG1*, *NOTCH3* and *HES1* were assessed by qPCR. (P) The protein expression levels of GDF15, β‐catenin, JAG1, NOTCH3 and HES1 in primary human EVTs were measured by Western blotting. Data are presented as the means ± SDs from at least three independent experiments. *p* < 0.05, and *p* < 0.01 by two‐tailed Student's *t* test.

Importantly, stable overexpression of JAG1 rescued the *GDF15* knockdown‐mediated reductions in cell migration and invasion in HTR‐8/SVneo cells (Figure 5A–F) and in primary human EVTs (Figure 5G–L). Moreover, the decreased NOTCH3 and HES1 expression levels after *GDF15* knockdown were partially rescued by JAG1 overexpression in HTR‐8/SVneo cells (Figure 5M,N) and in primary human EVTs (Figure 5O,P). However, JAG1 overexpression did not affect GDF15 or β‐catenin expression levels. These results (i) indicate that JAG1 functions downstream of GDF15 in regulating human EVT invasion and migration, while also (ii) implicating JAG1 in a mediating function for GDF15‐upregulated NOTCH3 and HES1 expression, thus defining a ‘GDF15–JAG1 axis’ in regulating human EVT invasion.

### 3.5 GDF15 decreases the embryo resorption rate in an abortion‐prone mouse model

**FIGURE 6:**
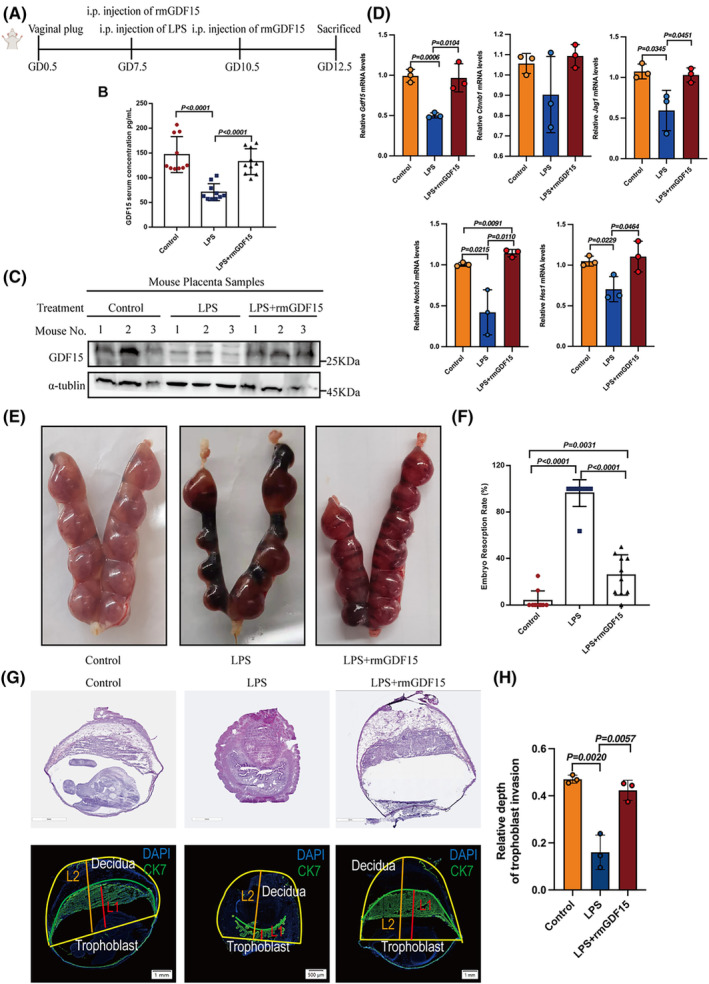
Decreased GDF15 level contributes toward the onset of abortion in mice. (A) Wild‐type pregnant mice were treated with LPS (0.25 mg/kg) or rmGDF15 (0.01 mg/kg) at gestational day (GD) 7.5, followed by supplementation with rmGDF15 at GD10.5. (B) GDF15 levels in the serum of mice from different treatment groups (*n* = 10 mice/group). (C) GDF15 protein levels in murine placentas from different groups (*n* = 3 mice/group). (D) *Gdf15*, *Jag1*, *Notch3* and *Hes1* mRNA levels in murine placentas from different groups (*n* = 3 mice/group). (E, F) Embryo resorption rates were examined in different treatment groups of mice with LPS‐induced abortion (*n* = 10 mice/group). (G, H) Depth of CK7+ trophoblast infiltration into the uterus of pregnant mice from the control, LPS or LPS + rmGDF15 groups (*n* = 3 mice/group) was observed by haematoxylin–eosin staining or immunofluorescence staining. Relative depth of trophoblast infiltration into the uterus: the ratio of CK7+ trophoblast depth (L1 in red) to the total depth (L2 in orange). Data are presented as the means ± SDs from at least three independent experiments. *p* < 0.05, and *p* < 0.01 by two‐tailed Student's *t* test.

To explore the potential beneficial effects of supplementation with GDF15 on early pregnancy maintenance *in vivo*, we examined the impacts of GDF15 supplementation in a classic LPS‐induced abortion mouse model (Figure 6A). Specifically, we examined whether GDF15 supplementation (0.01 mg/kg dose of recombinant murine GDF15, delivered via i.p. injection) decreased embryo resorption and/or protected against pregnancy loss. Initial analyses confirmed successful model induction, showing that LPS injection led to significantly decreased GDF15 level in murine serum and placentas (Figure 6B–D) and impaired the depth of trophoblast infiltration into the uterus (Figure 6G,H) while also promoting embryo resorption (Figure 6E,F). GDF15 supplementation of LPS‐induced abortion model mice increased circulating GDF15 level (*n* = 10/group; Figure 6B) and GDF15 mRNA and protein levels in murine placentas (*n* = 3/group; Figure 6C,D). Importantly, supplementation with rmGDF15 to LPS‐induced abortion mice led to significant decreases in the embryo resorption rate compared to the vehicle‐control mice (*n* = 10/group; Figure 6E,F).

To examine whether the observed decrease in embryo resorption upon GDF15 supplementation involves promotion of trophoblast infiltration into the uterus, we assessed the infiltration of placental trophoblast cells in an immunofluorescence analysis of the depth of CK7+ trophoblast infiltration into the uterus of pregnant mice. Indeed, compared to the vehicle‐control group, the GDF15‐treated model animals displayed significantly increased trophoblast invasion (*n* = 3/group; Figure 6G,H). Further, we tested whether low GDF15 level in an abortion‐prone mouse model were consistent with what was observed upon GDF15 knockdown in vitro. qPCR was performed in placentas and the results showed decreased *Jag1*, *Notch3* and *Hes1* mRNA levels in LPS‐induced abortion mouse model compared with the control (*n* = 3/group; Figure 6D). Beyond reinforcing our findings of a functional contribution from GDF15 in normal early pregnancy, these *in vivo* findings indicate that GDF15 supplementation apparently confers benefits for early pregnancy maintenance and could help reduce the risk of early pregnancy loss.

## 4 DISCUSSION

**FIGURE 7:**
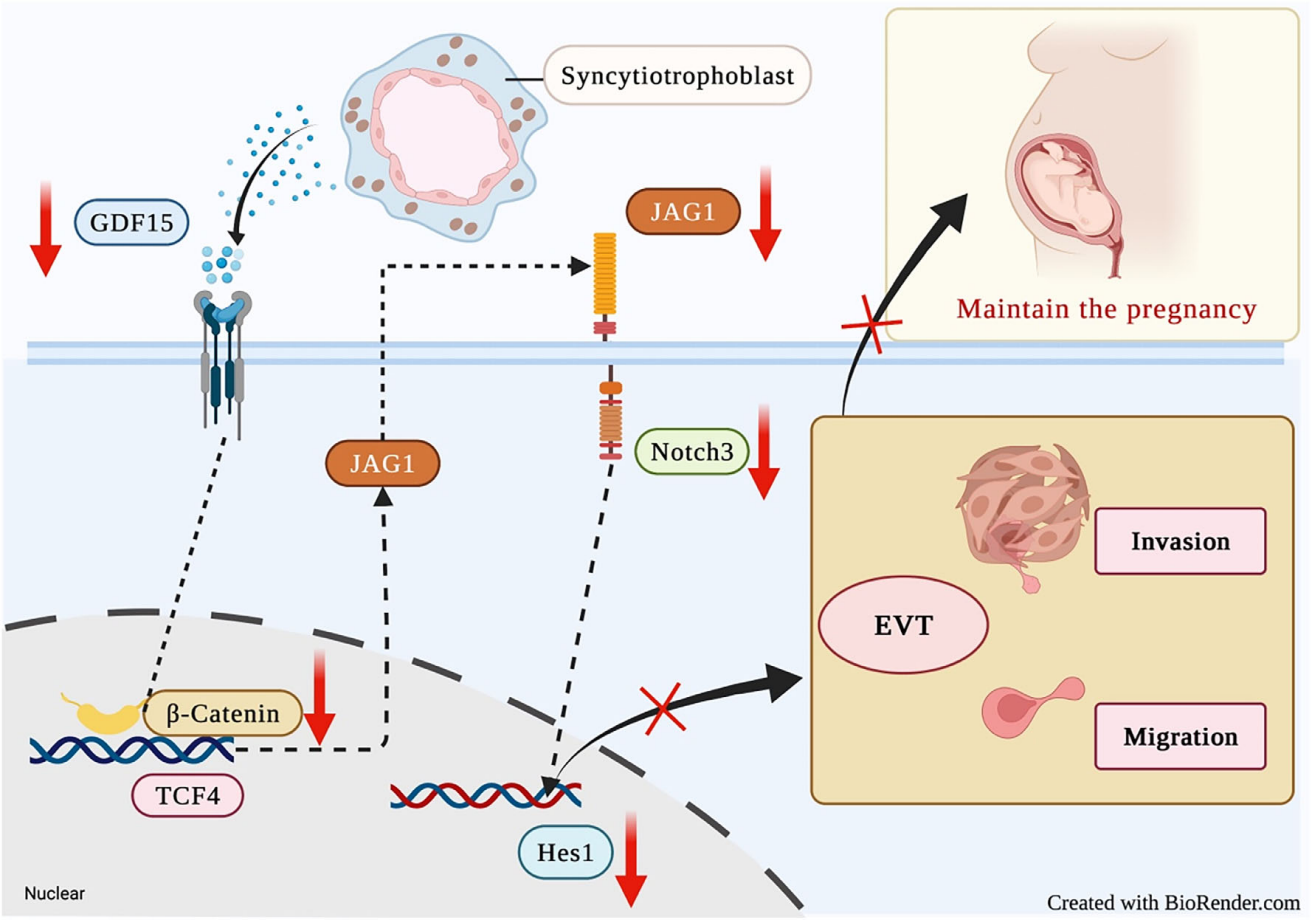
Schematic illustration indicating how the low embryonic villous GDF15 level increases URPL risk. Insufficient GDF15 expression contributes to the onset of URPL by decreasing EVT invasion via the suppression of JAG1/NOTCH3/HES1 signalling (red arrow). The figure was created with BioRender.com.

Multiple factors, including genetic abnormalities, immune dysfunction, smoking, and alcohol consumption, have been shown to contribute to the onset of RPL.^2^, ^4^ However, owing in part to its poorly understood pathogenic mechanism, the clinical management of URPL is still challenging. Insufficient EVT invasion is associated with several pregnancy complications, including pregnancy loss, preeclampsia, and foetal growth restriction.^14^, ^52^ Our results demonstrated that insufficient GDF15 expression impaired EVT invasion by suppressing JAG1/NOTCH3/HES1 signalling activity, thus linking GDF15 to URPL pathogenesis (Figure 7).

GDF15 was found highly expressed in the placenta during normal pregnancy which was believed to be produced by trophoblasts and decidual cells.^53^, ^54^, ^55^ Serum GDF15 levels increase with gestational age, but decrease markedly when abortion occurs.^31^, ^56^ Here, we found that the circulating and villous GDF15 levels were significantly decreased in patients with URPL compared to control pregnancies, adding an additional line of evidence that decreased GDF15 level during the first trimester of pregnancy are associated with URPL pathogenesis. We further found that GDF15 was abundantly expressed in the STBs and EVTs of first‐trimester villi compared to CTBs. Decreased GDF15 level were detected in STBs and EVTs in women with URPL compared to controls, suggesting a potential autocrine and/or paracrine role for GDF15 in regulating EVT cellular behaviours at the maternal‐foetal interface.

Sufficient EVT invasion into the maternal decidua is believed to be essential for endometrial spiral artery remodelling, placenta formation, and the maintenance of human pregnancy.^14^, ^57^, ^58^ TGF‐β superfamily members have been shown to exert a variety of functions in controlling trophoblast invasion, and have been proposed as candidate predictive and diagnostic markers for URPL.^56^ GDF15 participates in cancer cell invasion^43^, ^44^ and promotes epithelial‐to‐mesenchymal transition.^59^ In the present study, we identified GDF15 as a regulatory molecule in human EVT invasion and migration based on in vitro and ex vivo experimental models. Although our findings that rhGDF15 did not affect the apoptotic rates of HTR‐8/SVneo cells are inconsistent with a previous report,^27^ we note that this discrepancy between our findings and the previous findings could reflect the distinct durations of rhGDF15 treatment in HTR‐8/SVneo cells. Moreover, we confirmed our findings using an siRNA‐mediated *GDF15* knockdown approach in both HTR‐8/SVneo cells and primary human EVTs.

Dysregulation of the NOTCH ligand JAG1^60^ has been linked to pregnancy disorders such as preeclampsia^45^ and foetal growth restriction.^61^ Several studies have reported that the NOTCH signalling pathway promotes human EVT invasion and proliferation.^62^, ^63^, ^64^ In the present study, we found that *JAG1* knockdown decreased EVT invasion and migration. TGF‐β regulates cell differentiation, growth and embryonic development via NOTCH signaling.^65^ In mouse hepatic stellate cells, TGF‐β1 induces cell differentiation through regulation of JAG1/NOTCH signalling. Our results show that JAG1 functions downstream of GDF15. The decreases in EVT invasion induced by *GDF15* knockdown were rescued by JAG1 overexpression, providing evidence that JAG1 mediates the observed GDF15 knockdown‐mediated reductions in EVT invasion.

Several studies have reported that GDF15 can regulate cellular behaviours through β‐catenin signaling.^47^, ^48^, ^66^ JAG1 has been reported as a target molecule of β‐catenin in colorectal cancer,^49^ and JAG1 was shown to induce NOTCH3 expression in endometrial epithelial cells.^46^ It has been identified that GDNF‐family receptor α‐like (GFRAL), which is expressed at biologically meaningful levels in the hidbrain,^67^ acts as a receptor for GDF15 to exert biological functions in the central nervous system. Even though GFRAL is barely detectable beyond the central nervous system, we observed consistent results regarding the pro‐invasive role of GDF15 in human trophoblasts when we performed recombinant GDF15 treatment experiments and siTNA‐mediated GDF15 knockdown experiments. In addition, a recent paper has also reported a pro‐invasive role of GDF15 in human trophoblasts.^68^ It is conceivable that GDF15 could exert its effects through regulating other unidentified receptors or interfering other ligand‐receptor interactions. For example, outside the central nervous systems, GDF15 has been shown to suppress macrophage surveillance by activating canonical SMAD2/3 signalling and inhibiting TGF‐β‐activated kinase signalling to NF‐κB.^69^ In addition, GDF15 has been reported to promote cervical cancer cell proliferation by activating the PI3K/AKT and MAPK/ERK signalling pathways through the receptor ErB2.^55^ However, it is still unclear which receptor GDF15 acts on and whether it affects other ligand‐receptors in trophoblasts, which needs further investigation. In mammalian cells, *NOTCH1*, *NOTCH2*, *NOTCH3* and *NOTCH4* are transmembrane receptors that transduce signals of *DLL1*, *DLL3*, *DLL4*, *JAG1* and *JAG2*. It has been reported that knockdown of *JAG1* impairs endometrial receptivity and blastocyst implantation by reducing NOTCH signalling via NOTCH3.^46^ The binding of JAG1 to NOTCH1‐3 ultimately activates downstream pathways by inducing the transcription of target genes, including *HEY* and *HES*.^70^ Our findings demonstrated that aberrantly low GDF15 level suppressed JAG1, NOTCH3 and HES1 and lead to reduced β‐catenin accumulation.

A previous study reported that *GDF15*‐knockout mice are fertile,^71^ but the details of their fertility have not been reported. We found that supplementation with rmGDF15 protein decreased the embryo resorption rate in LPS‐induced abortion model mice, showing that GDF15 can exert a protective roles in pregnancy maintenance under specific adverse conditions. Of note, the ratio of L1/L2 cannot fully represent the invasiveness of trophoblasts in vivo; it can only indicate to a certain extent the depth of trophoblast infiltration into the uterus. In addition, placental samples from earlier timepoint than GD12.5 after LPS treatment have not been examined in our study, which need further investigation and may be more meaningful for evaluating impairment of early placental development.

## 5 CONCLUSIONS

In summary, our findings reveal that decreased GDF15 level in URPL can impair EVT invasion by suppressing the JAG1/NOTCH3/HES1 signalling pathway. We also identified GDF15 as a pro‐invasive factor in human EVT and a circulating biomarker for URPL occurrence. Our study offers an example for how identification of molecules and signalling pathways that support normal placental development can provide potentially valuable diagnostic and/or therapeutic targets for URPL clinical management, ideally thereby reducing the physical, psychological and financial burdens of URPL couples.

## AUTHOR CONTRIBUTIONS

Y.L. conceived and designed the project; CZ.L., TX.N., and TT.Z. performed the experiments and analysed the data; CZ.L. and YQ.G. collected the clinical tissue and serum samples; CZ.L. wrote the manuscript; Y.L., ZJ.C., and JH.Y. revised the manuscript. All authors read and approved the final manuscript.

## FUNDING STATEMENT

This work was supported by grants from the National Key Research and Development Program of China (2022YFC2702400), the National Natural Science Foundation of China (31988101, 82101784, 82171648), the Key Research and Development Program of Shandong Province (2021LCZX02), the Natural Science Foundation of Shandong Province (ZR2020QH051), and the Young Scholars Program of Shandong University.

## CONFLICT OF INTEREST STATEMENT

The authors have declared that no conflict of interest exists.

## Supporting information


**Data S1:** Supporting Information

## Data Availability

The RNA‐seq data generated in this study have been deposited at the Genome Sequence Archive for Human (https://ngdc.cncb.ac.cn/gsa-human) under accession numbers HRA002272 and HRA002274. Qualified researchers may apply for access to the original data pending institutional review board approval. All data generated or analyzed during this study are included in this article and its supplementary materials. Further information and requests for resources and reagents should be directed to and will be fulfilled by Junhao Yan (yyy306@126.com).
