# Supplementary material for: Insufficient GDF15 expression predisposes women to unexplained recurrent pregnancy loss by impairing extravillous trophoblast invasion
Source: Cell Prolif. 2023 Jun 4;56(12):e13514. doi: 10.1111/cpr.13514 (PMC10693185; doi:10.1111/cpr.13514)
Supplement: Supplementary file 1 — Data S1: Supporting Information [file CPR-56-e13514-s001.docx]

Supporting information for

**Insufficient GDF15 expression predisposes women to unexplained recurrent pregnancy loss by impairing extravillous trophoblast invasion**

Chunzi Lyu^1,2^, Tianxiang Ni^1,2^, Yaqiu Guo^3^, Tingting Zhou^1,2^, Zi-Jiang Chen^1,2^, Junhao Yan^1,2*^, Yan Li^1,2,4,5*^

^1^ Center for Reproductive Medicine, Shandong University, Jinan, Shandong, 250012, China

^2^ Key Laboratory of Reproductive Endocrinology of Ministry of Education, Shandong University, Jinan, Shandong, 250012, China

^3^ Department of Anesthesiology, Jinan Maternal and Child Health Hospital, Jinan, Shandong, 250000, China

^4^ Medical Integration and Practice Center, Shandong University, Jinan, Shandong, 250012, China

^5^ Lead Contact

^*^ Correspondence to: [yyy306@126.com](mailto:yyy306@126.com) and [ubcliyan@sdu.edu.cn](mailto:ubcliyan@sdu.edu.cn)

**This PDF file includes:**

Figure. S1 to S4

Table. S1


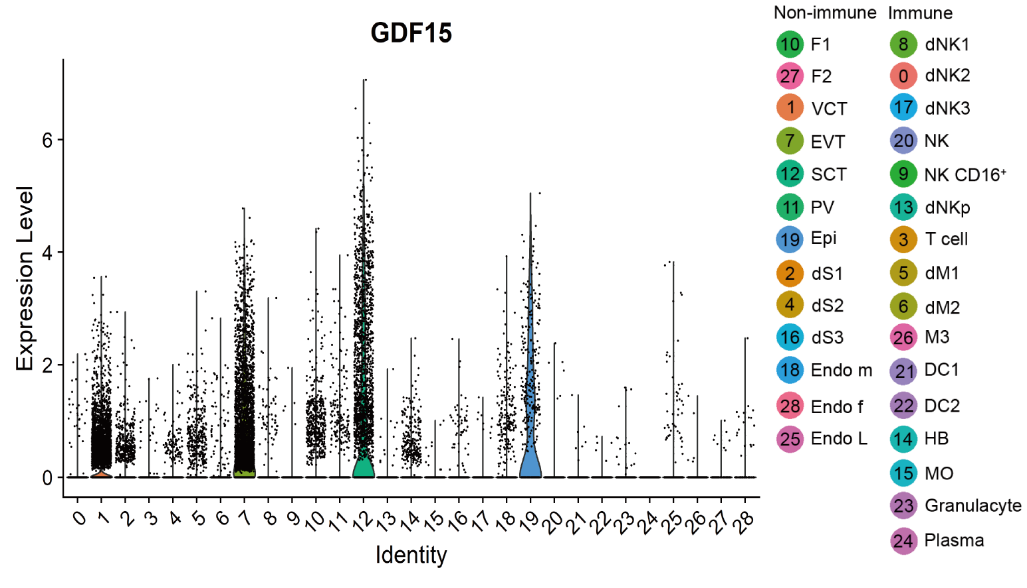


**Figure S1. GDF15 is expressed in EVT, VCT, and STB cells at the first-trimester maternal-fetal interface.** Feature plot and violin plot visualization of the log-transformed, normalized expression of GDF15 in different placental and decidual cell types from 10x Genomics and Smart-seq2 scRNA-seq analysis in published datasets (E-MTAB-6701 and E-MTAB-6678, RVento-Tormo et al., 2018). Colors indicate cell type. n=11 deciduas, n=5 placentas and n= 6 blood samples. F, fibroblasts; VCT, villous cytotrophoblast; EVT, extravillous trophoblast; SCT, syncytiotrophoblast; PV, perivascular cells; Epi, epithelial glandular cells; dS, decidual stromal cells; Endo, endothelial cells; NK, natural killer cells; dM, decidual macrophages; M3, maternal macrophages; DC, dendritic cells; HB, Hofbauer cells; MO, monocytes; d, decidual; m, maternal; f, fetal; l, lymphatic; p, proliferative.

**
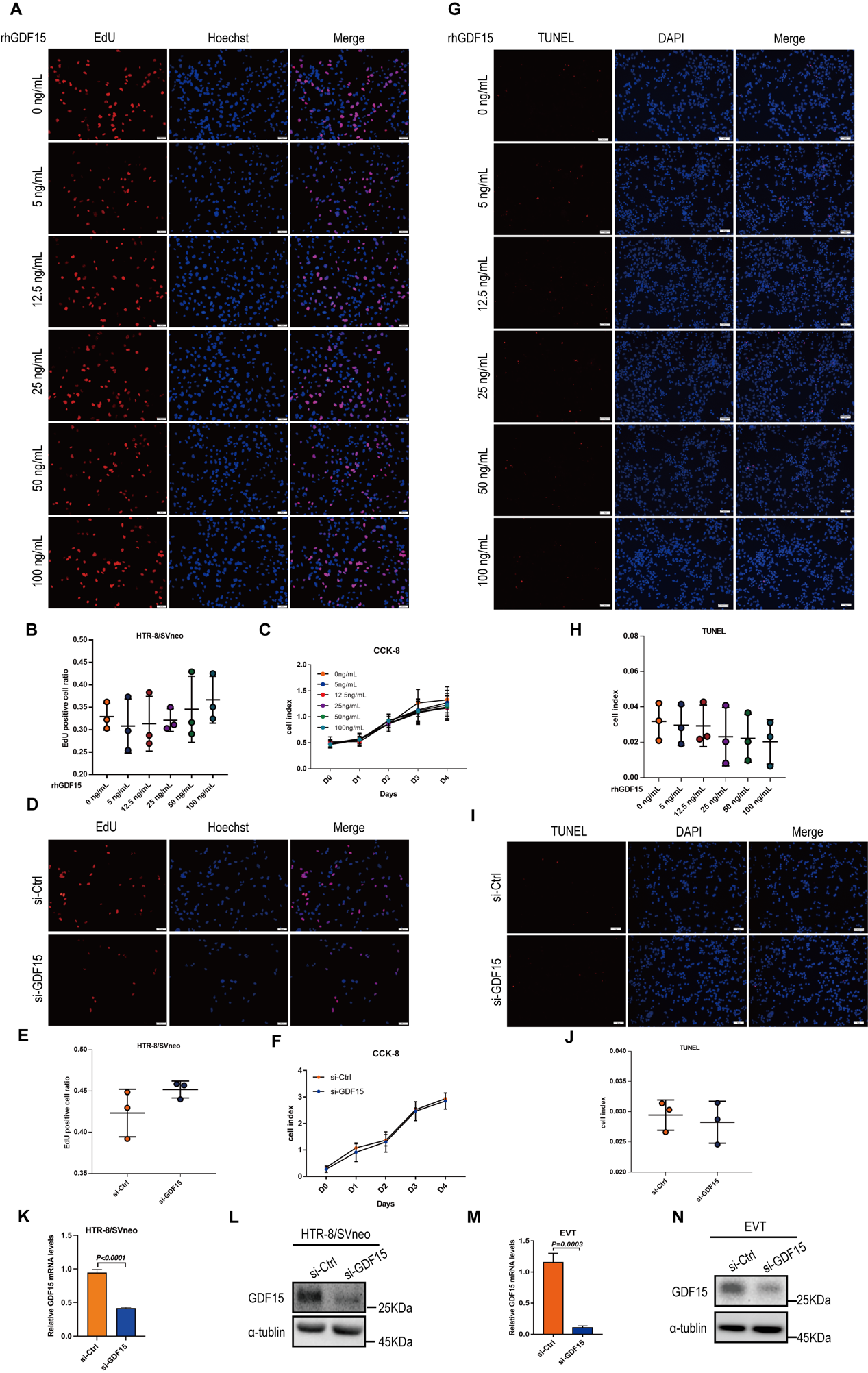
**

**Figure S2. GDF15 does not regulate human EVT proliferation or apoptosis.** EdU (**A-B**) and CCK-8 (**C**) assays were performed to examine the proliferation of HTR-8/SVneo cells treated with various concentrations of rhGDF15 (0 ng/mL, 5 ng/mL, 12.5 ng/mL, 25 ng/mL, 50 ng/mL, 100 ng/L). HTR-8/SVneo cell proliferation was examined with EdU assays (**D-E**) and CCK-8 (**F**) assays after transfection with non-targeting control siRNA (si-Ctrl) or siRNA targeting *GDF15* (si-GDF15). (**G-H**) TUNEL assays were performed to examine the apoptosis rates of HTR-8/SVneo cells treated with various concentrations of rhGDF15 (0 ng/mL, 5 ng/mL, 12.5 ng/mL, 25 ng/mL, 50 ng/mL, and 100 ng/L). The upper panel shows representative images of the TUNEL assay; the lower panel shows the summarized quantitative results of the TUNEL assay. (**I-J**) HTR-8/SVneo cell apoptosis was examined with TUNEL assays after transfection with or without si-GDF15. Representative images are shown in the upper panel; the summarized quantitative results are shown in the lower panel. qPCR (**K**) and western blotting (**L**) were performed to evaluate the GDF15-knockdown efficiency of siRNA transfection in HTR-8/SVneo cells. mRNA (**M**) and protein (**N**) levels of GDF15, without or with siRNA-mediated GDF15 knockdown, in primary human EVTs. Data are presented as the means ± SD of three independent experiments.


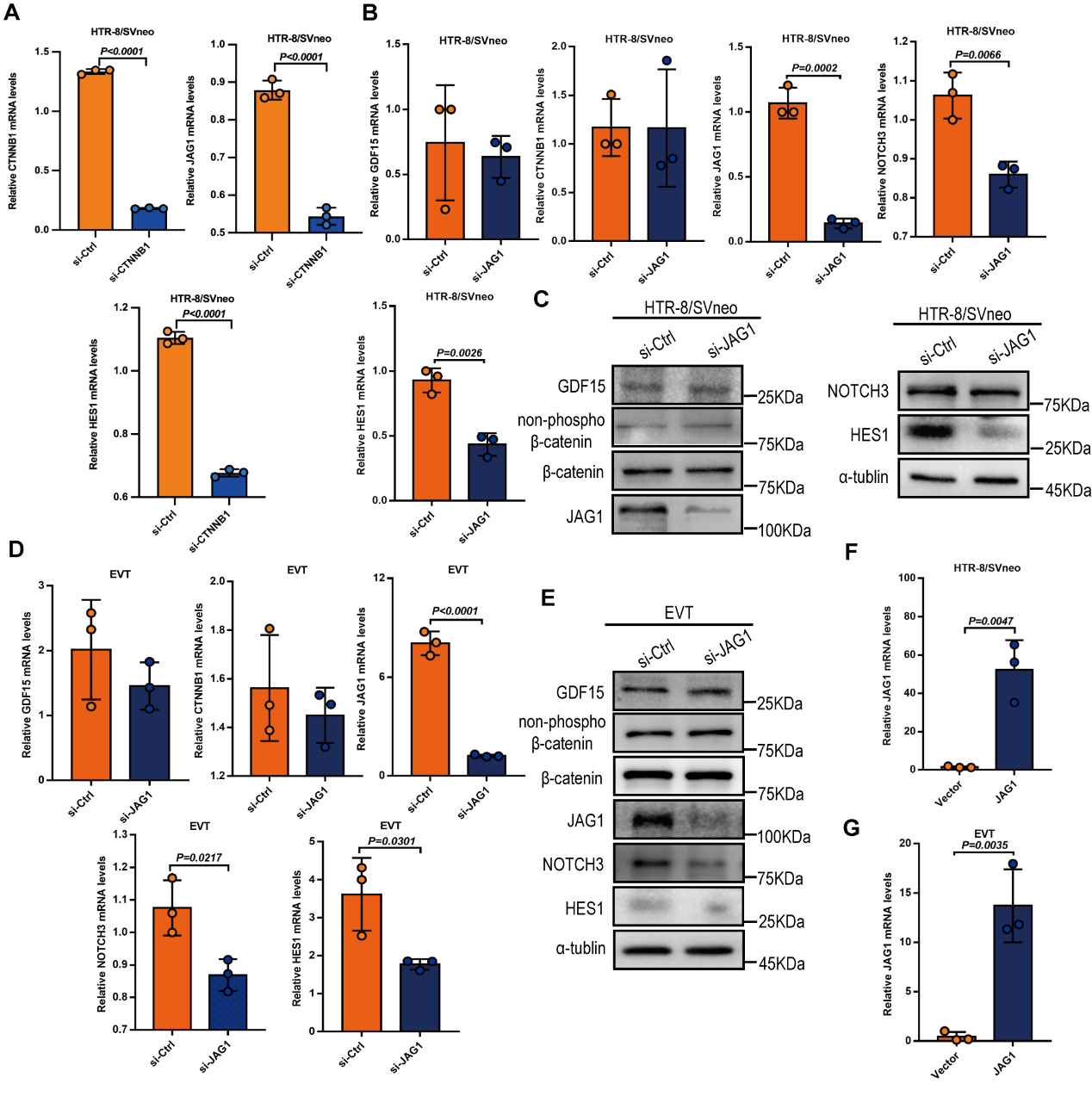


**Figure S3. JAG1 knockdown inhibits the NOTCH3/HES1 pathway.** (**A**) qPCR analysis of CTNNB1, JAG1, and HES1 transcriptional levels in HTR-8/SVneo cells transfected with si-Ctrl or si-CTNNB1. (**B**) qPCR analysis of GDF15, CTNNB1, JAG1, NOTCH3, and HES1 mRNA levels in HTR-8/SVneo cells transfected with si-Ctrl or si-JAG1 for 48 h. (**C**) Western blotting analysis of GDF15, β-catenin, JAG1, NOTCH3, and HES1 protein levels in HTR-8/SVneo cells transfected with si-Ctrl or si-JAG1 for 48 h. qPCR (**D**) and western blotting (**E**) analysis of GDF15, β-catenin, JAG1, NOTCH3, and HES1 expression in primary human EVTs transfected with si-Ctrl or si-CTNNB1. (**F-G**) The mRNA levels of JAG1 overexpression in HTR-8/SVneo cells and primary human EVTs. Data are presented as the means ± SDs from at least three independent experiments. p<0.05, and p<0.01 by two-tailed Student’s t test.

**
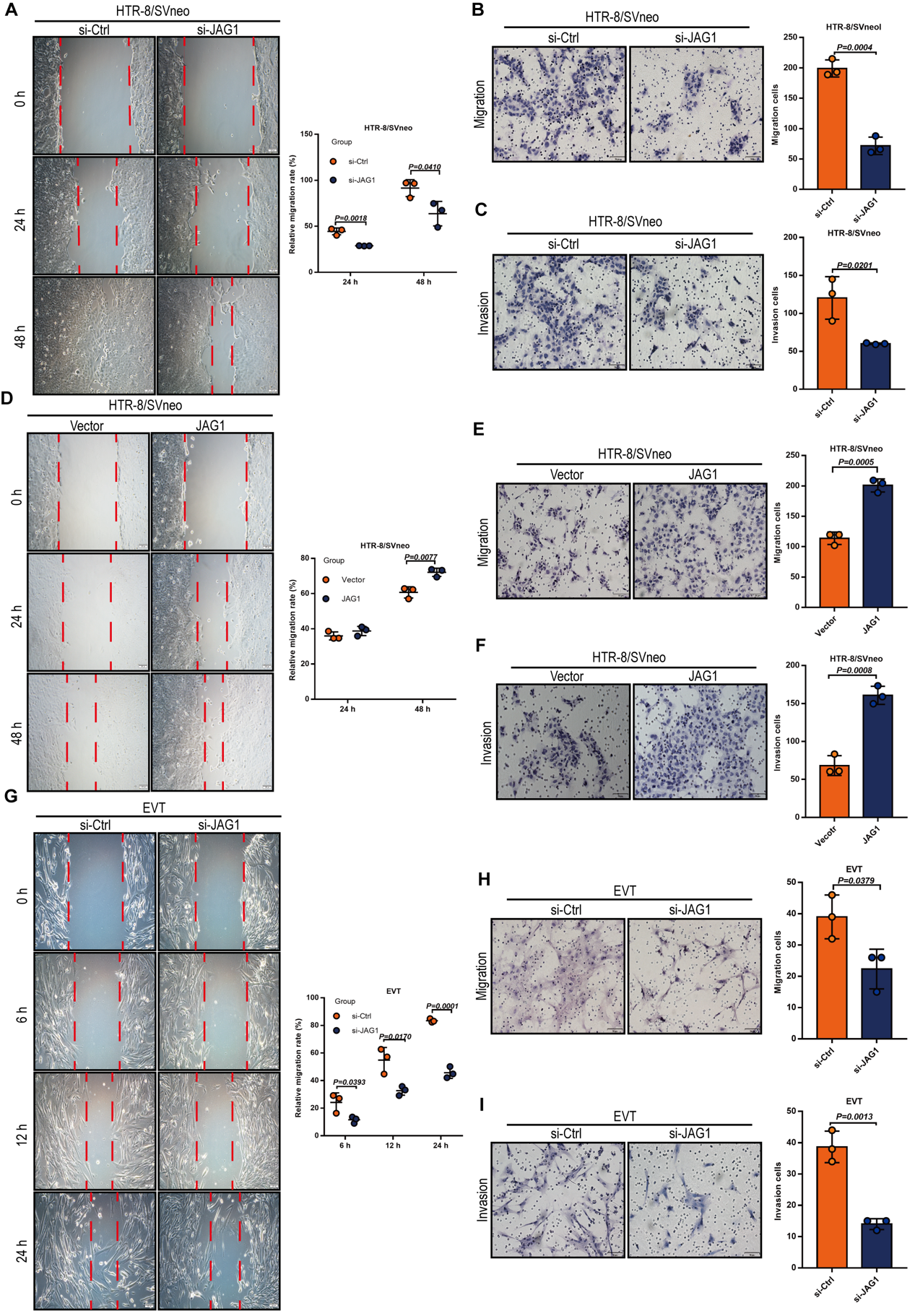
**

**Figure S4. JAG1 promotes invasion, and migration of human extravillous trophoblasts.** HTR-8/SVneo cell mobility was examined with wound-healing assays (**A**) and Transwell™ assays (**B**) after transfection with non-targeting control siRNA (si-Ctrl) or siRNA targeting *JAG1* (si-JAG1). (**C**) Cell invasiveness was examined with Matrigel-coated Transwell™ assays. Wound-healing assays (**D**) and Transwell™ assays (**E**) were performed to examine the migratory abilities of JAG1-overexpressing HTR-8/SVneo cells. (**F**) Matrigel-coated Transwell™ assays were performed to study cell invasion in HTR-8/SVneo cells after transfection with or without JAG1-expressing adenovirus. Primary human EVTs were transfected with si-JAG1, followed by wound-healing assays (**G**) and Transwell™ assays (**H**) of cell migratory capacity and Matrigel-coated Transwell™ assays of cell invasiveness (**I**). Data are presented as the means ±SDs from at least three independent experiments. p<0.05, and p<0.01 by two-tailed Student’s t test.

**Table S1. qPCR primer sequences**

| **Primer name** | **Sequence (5’ → 3’)** |
| --- | --- |
| Homo-GDF15-103F | GCTACGAGGACCTGCTAACC |
| Homo-GDF15-103R | ACTTCTGGCGTGAGTATCCG |
| Homo-JAG1-195F | CGGGAAGTGCAAGAGTCAGT |
| Homo-JAG1-195R | TTGGTTTCACAGTAGGCCCC |
| Homo-CTNNB1-155F | GCTGCAACTAAACAGGAAGGG |
| Homo-CTNNB1-155R | CCCACTTGGCAGACCATCAT |
| Homo-Notch1-180F | GCAAGAACGCCGGGACA |
| Homo-Notch1-180R | GGCACTTGTACTCCGTCAGC |
| Homo-Notch2-156F | GATGACTGCCCTAACCACAGG |
| Homo-Notch2-156F | GCCCCCATTTTGACAGGCAT |
| Homo-Notch3-190F | CGTCAGTGTGAACTCCTCTCC |
| Homo-Notch3-190F | CCAGGTTGGTGCAGATACCAT |
| Homo-Hes1-155F | ACACGACACCGGATAAACCAA |
| Homo-Hes1-155R | GGAATGCCGCGAGCTATCTT |
| Homo-β-actin-191F | GAAGAGCTACGAGCTGCCTGA |
| Homo-β-actin-191R | CAGACAGCACTGTGTTGGCG |
| Mus-actin-154F | GGCTGTATTCCCCTCCATCG |
| Mus-actin-154R | CCAGTTGGTAACAATGCCATGT |
| Mus-Gdf15-124F | GCAATGCCTGAACAGCGAC |
| Mus-Gdf15-124R | CTGAGTTCGAGTCCTCTCGG |
| Mus-Jag1-118F | GGGATGATGGGAACCCTGTC |
| Mus-Jag1-118R | GGACGCCTCTGAACTCTGAC |
| Mus-Ctnnb1-110F | TTGTAGAAGCTGGTGGGATGC |
| Mus-Ctnnb1-110R | AGTCGCTGCATCTGAAAGGT |
| Mus-Notch3-98F | TTCCCCGTGTCGTAATGGTG |
| Mus-Notch3-98R | TGACTTCACAGTTCTGGCCC |
| Mus-Hes1-138F | GCGTGTTGGGGAAATACCG |
| Mus-Hes1-138R | AGATCTGGGTCATGCAGTTGG |
